# Meeting Review: The Intelligent Systems in Bioinformatics Conference 2001 (ISMB2001)

**DOI:** 10.1002/cfg.108

**Published:** 2001-10

**Authors:** K. Cara Woodwark

**Affiliations:** Biomolecular Sciences UMIST, PO Box 88 PO Box 88 Manchester M60 1QD UK


**Comparative and Functional Genomics**
*Comp Funct Genom* 2001; **2**: 330–337.
**DOI:** 10.1002/cfg.108

**Feature**

### Meeting Review: The Intelligent Systems in Bioinformatics Conference 2001 (ISMB2001)

21st–25th July, Tivoli Gardens, Copenhagen

K. Cara Woodwark*
*Biomolecular Sciences, UMIST, Manchester, M60 1QD, UK*

*Correspondence to:
K. Cara Woodwark, Biomolecular Sciences, UMIST, PO Box 88, Manchester, M60 1QD, UK.
E-mail: kcw@bms.umist.ac.uk*

**Keywords: bioinformatics; conference; microarray; promoter; promoter prediction; gene prediction; RNA**

This year's ISMB conference, an annual event organised by the International Society for Computational Biology (**http://www.iscb.org**) was the biggest ever, with over 1400 delegates. The venue was also a first, situated, as it was, in Copenhagen's Tivoli Gardens funfair. However there was still time for the many satellite meetings that flanked the conference including the Bio Pathways conference, the Bioinformatics Open Source Conference and the Bio-Ontologies Conference.

**Soren Brunak** and **Anders Krogh** opened the conference. They remarked that it was 30 years since the Needleman-Wunsch algorithm was written, but that things have not changed much, from that time, in that the basic biological ideas are still driving research. They also mentioned that there had been 180 papers submitted to the conference all of which had to be refereed and graded before choosing the 38 speakers.

Only the keynote talks are covered in depth here as all the other talks are covered in a special supplement to the journal Bioinformatics. **http://bioinformatics.oupjournals.org/**

The conference began with talks on **Protein Structure and Modelling**. However, rather than talks about protein structure prediction, these talks were based much more on the biology of how protein structure information can help our understanding of the evolution and function of proteins.

**Chris Dobson** (Cambridge University) opened the conference with an excellent talk on *Protein Folding, Molecular Evolution, and Human Disease*. He explained that, given the right circumstances, any protein will form fibrils similar to those found in BSE or Alzheimer's.

Proteins seem to fold and unfold all the time, which can cause problems for structure determination in protein crystallography and NMR, as proteins made under different circumstances often have different structures. In the densely packed environment of the cell, folding and unfolding may form part of a switch mechanism, or chaperones may be involved to help a protein to fold into a particular structure.

Aggregations or amyloid structures are responsible for many diseases e.g. Alzheimers, New variant CJD (related to BSE), Type II diabetes etc. Apparently, by the age of 60 we will all develop some sort of aggregate, but hopefully they should be disease free (asymptomatic). 16 diseases caused by amyloid structures have now been identified (20 if diseases such as Parkinsons are included). For example, two point mutations in Lysozyme allow it to form disease-causing fibrils, composed of many parallel beta sheets.

The major breakthrough came when one of Chris' students was working on PI3 Kinase NMR, when he went for a long weekend (160 hours). On his return, the trace had disappeared almost to nothing. So they looked to see what had happened to the protein and found that it had formed fibrils. This was a complete surprise as it wasn't a disease causing protein and so was not expected to form fibrils. After examining the fibrils they discovered

Copyright © 2001 John Wiley & Sons, Ltd.

that they were hollow pipes formed by 4 groups of 2 beta sheets wrapped around each other in a helical formation. These might be useful as nanotubes!

Chris believes that the ability to form fibrils is a character of all proteins, for example, even myoglobin, in its less soluble form, produced amyloid fibres. In fact all proteins they have tried have produced fibrils, given the correct circumstances, and any polypeptide chain if not chaperoned, or controlled, could form fibrils.

There appears to be an initial time limiting step, as, like crystallisation, fibril formation needs a nucleation, or seeding, step. This explains the rapid onset of diseases such as BSE after the first symptoms are noticed, as after the initial contamination with "seed" proteins there is a slow "incubation" period until enough plaques are formed to cause symptoms. After these first signs the growth of the fibrils takes place rapidly, especially as the intermediate form of the fibril is the most "contagious". Initial aggregates rather than the fibrils are the real seeds. At this stage they are toxic and may lead to apoptosis, thus ridding the body of a diseased cell, although this is not always a good thing, as even more "seed" forming fibrils may be released, to be taken up by other cells.

The reason that age seems to be a factor in many of these amyloid diseases is that over the years there is an increased risk that something will go wrong with the folding of a protein, thus forming a nucleation seed. However, some proteins are more likely to aggregate than others, due to sequence and cellular circumstance. For example, some mutated proteins aggregate faster than others from a single point mutation. If, for example, the mutation is in the C terminus, then it is more likely to cause protein aggregation, even though it may not have much affect on the folding. The position and "biology" of a protein is also important, as for example, *in vitro*, myoglobin will aggregate more quickly than prions, yet there is no myoglobin aggregate disease. Therefore, whether a protein forms aggregates also depends on its position and interactions within the cell. Selection against aggregation may have increased the occurrence of chaperones, as protein mixes do not form fibrils. Heterozygotes, with two alleles of a protein, may also be at an advantage as mixed fibrils are also less likely, so even sexual reproduction plays a role. Chris believes that the whole of evolution and biology is one huge strategy to keep organisms from becoming massive balls of fibrils as if we were to live long enough that is how we would end up!

All of this was discovered because someone had a long weekend!

The rest of the section was an interesting mix of different aspects of protein structure. **Gordana Apic** (MRC Laboratory of Molecular Medicine) gave us an *Insight into Domain Combinations*. Potentially there are 180 000 pairwise combinations of SCOP domains, but only 1,000 of these are found in 20 000 multidomain proteins from 40 species. Indeed, 60% of domains have only one known combination partner. The domain order is highly conserved within protein families. **Stephen Möller** (EBI) spoke about predicting not only G protein coupled receptors, but also their specificity, using "SPEXS" (**http://ep.ebi.ac.uk/, http://www.ebi.ac.uk/~croning/coupling.html**). **Gianluca Pollastri** (UC Irvine & Bologna) used bi-directional neural network architectures and evolutionary information to predict interaction positions between proteins, as structure tends to be more conserved than sequence (**http://promoter.ics.uci.edu/BRNN-PRED/**). **Tobias Müller** (Deutsches Krebsforschungszentrum) also combined structure prediction with sequence searching, but this time used transmembrane domain specific matrices in order to facilitate the search for homologous transmembrane proteins (**http://www.dkfz.de/tbi/people/tmueller**). **Michael Lappe** (EBI) used a combination of structure, in the form of fold information, and protein interaction data to predict function, although the method is still very much in development (**http://www.ebi.ac.uk/~lappe/FoldPred**).

**Chris Sander** (Whitehead Institute) gave the keynote talk for the **Sequence Motifs, Alignments and Families** Section. He introduced his talk on *Structural Genomics*, by pointing out what was to be the overwhelming take-home message of the conference – that what we (Bioinformaticians as well as Biologists) are trying to do is answer Biological questions. He hoped that soon we would be able to model the 'e-cell' and then the 'e-organ'; model the perturbation of the system caused by drugs; decipher neurobiology, and combine it all into systems biology where not just the whole organism, but whole ecosystems could be modelled.

According to Chris Sander, structural genomics is only a part of what is needed. Eventually he would like to be able to see the structure of every biological molecule, from which we would be able to fully understand the function of that molecule.

Copyright © 2001 John Wiley & Sons, Ltd. 

However, protein structure is difficult and time consuming to obtain. Sander suggests that rather than obtaining the structure of every molecule, we need only determine the structure of one molecule per protein family, or maybe even superfamily, using homology to predict the structure of the other family members.

Even at the level of 30% sequence identity, there are 4000 sequences for all the families of the model organisms in Pfam. However, not all families are represented in Pfam, so the actual number may be closer to $4000 \times 4$. Producing this number of representative structures will take as much coordination as sequencing the human genome and to ensure availability to everyone, something that Bill Clinton and Tony Blair agreed to, it will involve much public funding. At present there are 7+ pilot centres that have started work, with other teams trying to predict function from structure (e.g. if the substrate size can be predicted from the substrate binding site, this narrows down the potential functions the molecule may have), which can then be tested in the lab.

The second part of the talk (Cell) concerned linking expression profiling with pathway databases, so that genes are not clustered just by expression, but by position in a pathway. Producing a functional distance pathway averaged into a neighbour pathway makes it possible to pick out "activity centres" of clusters of genes. As an example, Chris mentioned MYC, an enzyme that did not share a transcription profile with other enzymes from the same pathway, but which formed the nucleus of an activity centre. It was found that this enzyme was regulated by methylation, showing that enzymes within the same functional pathway may have different methods of regulation.

**Michael Wise** (CCSR, Cambridge, UK) continued the session by championing low complexity proteins, which are often ignored, as they are difficult to deal with. However, they still have function, for example those involved in DNA binding. He showed a method for modelling the low complexity regions based on pattern matching methods and was then able to categorise different types according to structure and function.

The other three talks in the session all dealt with regulation. **Mathieu Blanchette** (University of Washington) presented an interesting promoter prediction technique based on $X^2$ comparisons of the number of expected occurrences of a sequence over the number of sequences found. **Fabian Model** (Epigenomics AG) differentiated between acute lymphoblastic leukaemia (ALL) and acute myeloid leukaemia (AML) by looking at the methylation state of CpG dinucleotides in CpG islands, which are responsible for expression regulation. Their algorithm enabled the most discriminant sites to be chosen. **Giulio Pavesi** (University of Milan) explained the huge numbers involved in promoter prediction. For example, if a pattern of m letters is in all sequences studied and may have mutations at any position, then there are $4^m$ possibilities. While promoter regions are very small, e.g. $m=6$, this is a tractable number, 4096 possibilities, but with a signal length of 20, then it is in to the trillions (109,951,1627,776 to be exact). He used suffix trees to find planted promoter regions in simulated data, but the method has not yet been tested on real data.

Monday began with **David Eisenberg** (UCLA) giving the keynote talk for the **Networks and Modelling** section, with a talk on *Protein Interactions*. David explained that there were two types of protein interaction, physical and functional. Physical interactions are where two proteins come into actual contact. The Database of Interacting Proteins (DIP) found at **http://dip.doe-mbi.ucla.edu** contains details of interactions such as those elucidated from yeast 2-hybrid data, as well as data from literature searches. At present, yeast has the largest physical interaction network in the database, with *Helicobacter pylori* in second place. In yeast, a protein may be involved with as many as 50 interactions, although the average is 3.5. The highly connected genes are usually lethal, if deleted! Surprisingly, there is very little overlap detected so far in the physical interaction maps obtained from different organisms, but these are by no means complete yet.

Functional interactions group together proteins that are from the same pathways. Of course, proteins in functional groups can also be in the same physical group. Two methods of predicting whether genes are from the same pathway are microarray clustering and phylogenetic clustering. The two methods are similar, but phylogenetic clustering, instead of looking to see if genes are expressed in a similar pattern, looks to see if genes are inherited in similar pattern, as genes from the same pathway will tend to be inherited together. A continuation of phylogenetic inheritance clustering is the concept of the "gene neighbour". This is especially true in prokaryotes, where genes found in the same region of the chromosome, and therefore

Copyright © 2001 John Wiley & Sons, Ltd.

usually inherited together, are often functionally connected. Taking this a step further gives us the 'Rosetta Stone' situation, where proteins that interact and are separate in some species have fused into one protein in another species. In yeast, many single proteins have been found that have corresponding Rosetta Stone proteins in other species.

**Vincent Schäcter** (Hybrigenics) was interested in predicting Protein Interaction Maps (PIM) for one species based on what is known for a closely related species. This appeared to work reasonably well, but, as is often the case, a true validation dataset is unavailable.

**Dana Pe'er**, (Hebrew University) who won the award for best presentation, provided another approach to predicting interaction networks, in this case metabolic, signalling and regulatory networks. Their approach to *Inferring Subnetworks from Perturbed Expression Profiles* was to compare expression profiles from *Saccharomyces cerevisiae* knock out mutants with those from the wild type, under different perturbations, in order to determine which genes are affected by the deleted gene.

First they adaptively discretize the expression data into under expressed, baseline, and over expressed, for each gene. Bootstrap analysis is then performed to estimate the confidence of the features of the Bayesian networks. This gives levels of significance and confidence to the various pairwise interaction models, for example A upregulates B. From these pairwise models, interactions with high levels of confidence are extracted and grouped into subnetworks, representing cellular processes, such as iron homeostasis, or amino acid metabolism. These can then be visualized using "Pathway Explorer". They emphasize that they are unable to uncover all pathways this way but that those they have predicted may help the bench scientist to focus their research. For more information see **http://www.cs.huji.ac.il/labs/compbio/ismb01**.

**Laurence Hunter** (National Cancer Institute) tried a Bayesian method to improve clustering of microarray data but was unable to prove any increase in performance, possibly due to the small size of test data sets. **Eran Segal** (Stanford) integrated multiple datatypes including function, and known promoters, to help cluster microarray data. They found an interesting set of 17 yeast genes induced by the diauxic shift which are all involved in sugar metabolism and which all have two or more binding units for the MIG1 repressor. It had not previously been known that these genes were regulated by MIG1 (**http://dags.stanford.edu/bio**). **Chen-Hsiang Yeang** (MIT) was able to classify tumour types based on tissue specific gene regulation by "one versus all support vector machine" classification (**http://www.genome.wi.mit.edu/MPR/**). **Alexander Zien** (German National Research Center for Information technology) described a method called "Centralization" for comparing microarray experiments, based on the assumption that unless a cell is undergoing activation, then approximately the same number of genes will be upregulated as down regulated. This assumption appears to hold true over most cells as activation only occurs in a few cell types in the body such as stem cells.

**Bernardo Huberman** (Hewlett Packard) gave an interesting keynote talk on *The Phenomenon of the World Wide Web*. The web is now growing exponentially with at present almost 30 million web sites. In 1998 only 147 million people used the web, by 2000 this figure had grown to 400 million. He showed a map of all the web sites in Finland and how they interact. This map appears to be truly random but a similar random map could be used to represent Europe or America, as the rules, which can be drawn from this map, can be applied to any site linkage map. For example, the average number of links between any 2 pages in the Finnish web map is 4.2, the same as for any two pages in the whole of Europe.

He introduced a Social Science approach to searching the web. Social Science is concerned with the links between people, such as who knows whom on a University campus. A similar approach can be taken with the www in that if we want to know something we would ask someone who might know, and if they don't know then they ask someone else until the question is answered. If we were to search the web using the same approach we would ask a website concerned with the area we are interested in, that had many links. It will then ask all the sites it has links with until the question is answered. With a web of 10,000 sites, 50% of the web would be covered in just 12 steps. More details are available from **http://www.hpl.hp.com/shl**

Continuing in the promoter prediction theme **Derek Chiang** (UC Berkeley) told us of a method of looking for 5, 6, 7 and 8mers in the upstream regions of genes, then calculating a mean expression profile for all the genes with that motif. If this value differs significantly from the mean taken over all the genes, the motif may be a promoter. They have

Copyright © 2001 John Wiley & Sons, Ltd.

found some yeast promoters this way and are investigating many more potential promoters (**http://rana.lbl.gov**). **Anja von Heydebreck** (Max-Planck-Institute for Molecular Genetics) and **Eric Xing** (UC Berkeley) were both trying to differentiate between AML and ALL using microarrays. Both used two-step procedures. Heydebreck used a bipartite statistical analysis on only the highly expressed genes and was able to differentiate between AML and ALL, but also between the B and T forms of ALL. Xing's method involved clustering all the data, choosing the genes which were the most discriminatory and reclustering these, with the last 2 steps being iterated up to 9 times. He was also able to differentiate between AML and ALL. Interestingly both methods found three exceptions.

Tuesday morning saw the highlight of the conference for me, which was the keynote talk for **Gene Structure, Regulation and Modelling** by **Sean Eddy** (Washington University) on *The Modern RNA World: Many Genes don't Encode Proteins.* He started by mentioning that the competition for the number of genes in the human genome (GeneSweep), run from the Cold Spring Harbor Laboratory, is only for the number of protein coding genes, as Ewan Birney had felt that the RNA genes would be too difficult to assess. He also mentioned the yeast paper "Life with 6,000 genes" by Goffeau *et al.*, as this value does not include the many RNA genes, such as the 275 tRNA genes. However, RNA genes should not be underestimated, as a *C. elegans* RNA of only 21 nucleotides is lethal when deleted and is 100% conserved in mouse and *Drosophila*. Sometimes, in what would appear to be an ordinary looking gene with exons and introns, we find that the exons are not conserved but the introns are. In the case of the human UHG (U22 Host gene) "inside-out" gene, the introns encode 8 stable snoRNAs, which are spliced from the mRNA, while the resulting exons are quickly degraded (Figure 1, Tycowski *et al.*, 1996).

RNAs can also play vital roles in pathways, such as pRNA in bacteriophage $\phi 29$, which forms part of the phage rotary packaging motor. RNA genes may even be disease genes; Prader Willi syndrome and Cartilage–hair Hypoplasia are both caused by mutations in RNA sequences. In the latter case, they only discovered it was an RNA that was responsible when they had narrowed down the region on the chromosome to a short region in which it was known that none of the protein coding genes to be involved. However, an RNA gene (that had already been sequenced for another reason) was noticed and experiments showed that this was the responsible gene.

RNA genes are often difficult to detect as they are very small (such as the *C. elegans* RNA mentioned earlier); sometimes multicopy and redundant; often not polyadenylated (EST libraries are selected by poly-A); immune to frameshift errors or nonsense mutations; have no open reading frame or codon bias and often evolve rapidly.

Two recent screenings of *E. coli* have found 14 and 17 novel RNAs by either looking for promoters and conserved regions between closely related species, or by looking for conserved regions, then using microarrays to detect if they are expressed. Sean Eddy's group have also screened *E. coli*, finding another 7 novel RNAs not found by the other methods, with another 200+ potential RNAs still to be evaluated. This method involved comparing *E. coli* to four other bacterial genomes using Washington University's BLASTn, then running them through their QRNA program, to predict possible conserved RNAs.

QRNA is based on context free grammars (CFG), which are used to look for conserved secondary structure pairings in the BLAST alignments. Context free grammars look at nested pairwise correlations and can model all RNA secondary structures except pseudoknots, where the RNA crosses over on itself. QRNA appears to be a useful tool in the hunt for RNAs but does need several whole genomes, from species closely related

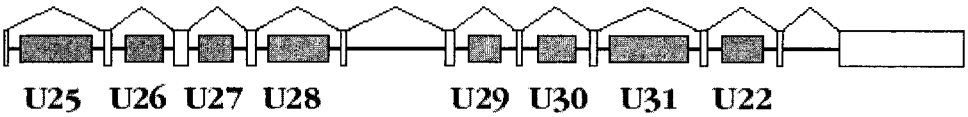

**Figure .** Inside-out genes? Human UHG (U22 host gene, white boxes) has no significant ORFs, is not conserved with mouse and is rapidly degraded. However, it has eight intron-encoded snoRNAs (shaded boxes), which are conserved with mouse and are stable (Tycowski *et al.*, 1996)

Copyright © 2001 John Wiley & Sons, Ltd.

to the species of interest, to be able to detect the conserved RNAs.

Sean Eddy left us with his vision of the modern RNA world where RNA may be the material of choice for cells, as small, highly specific, complementary RNAs are easy to produce by simply duplicating a region of the antisense strand of the target RNA, whereas to produce a protein to do the same job would take years of evolution and would be much less cost effective to the cell.

**Sridhar Hannenhalli** (Celera Genomics) was also interested in promoter prediction but without the help of microarray data. In humans almost half of all genes and nearly all house keeping genes have CpG islands in the promoter regions. **Uwe Ohler** (University of Erlangen-Nürnberg) used physical properties of DNA in his hunt for promoters. Properties such as propeller twist and DNA bending stiffness may be calculated from trinucleotide frequencies. In *D. melanogaster* known promoters are found with an accuracy of 75% using this method (**http://www.fruitfly.org/sequence/drosophila_datasets.html**). **Ammos Tanay** (Tel-Aviv University) introduced the concept of Genetic Networks, which describe the interactions, such as promotion or inhibition, between genes. By expanding networks based on gene expression profiles they were able to predict a novel yeast ergosterol transcription factor. **Carol Friedman** (Columbia University, New York) used Natural Language Processing (NLP) based on MedLEE, an existing medical NLP system, to extract information on molecular pathways from whole articles. Although the method did not find all the relationships that a human expert found it did find some real relationships that the human had missed. On a rather different note **Steven Skiena** (State University, New York) described an algorithm for *Designing better Phages*. This reduces the number of restriction sites by exploiting the redundancy inherent in the triplet code, so as to increase the bacteriophages use as an antibacterial agent.

**Chris Burge** from MIT presented the second Chris Overton memorial lecture and was presented with the Chris Overton Medal for Research by Chris Overton's widow. Christopher Burge is best known for producing Genscan as part of his PhD. His talk, entitled the *Computational Analysis of RNA Splicing*, explained that there is still much that we do not know about intron splicing and the regulation of alternative splicing, which appears to be very important in the human genome, where at least half of genes may be alternatively spliced.

There are two main intron types. Those where the splicing instructions are in the ends of the exons (which can lead to exon slippage) and is the usual method for human long intron splicing and those which splice short introns where the information is found in the intron ends. In worm and fly these last type are fairly constant, with an intron prediction accuracy of 95%, although the position of the 3′ splice site can wobble. In humans and plants there is much more variation in the intronic splice site elements, so that intronic enhancers become more important for accurate splicing. These are U rich in *Arabidopsis*; GGG or U rich in human, and UA, U or UGA rich in *Drosophila*. If the splice sites are strengthened then these intronic enhancers become unnecessary. He is currently developing computational methods to predict intronic splicing enhancers.

**Vasileios Hatzivassiloglou** (Columbia University) revisited Natural Language Processing, with a machine learning approach, also based on MedLEE. This method is able to not only extract relationships between proteins and genes but due to the large corpus of text, it can check and correct these relationships. **John Chuang** (University of Illinois) also used a method based on NLP, but to predict genes. Potential start, stop, donor and acceptor sites are scored and converted into Directed Acyclic Graphs (DAGs). The shortest path through the graph represents the predicted gene structure. **Ezekiel Adebiyi** (University of Tübingen) introduced an algorithm for finding approximate non-tandem repeats. These are difficult to find, due to insertions, deletions and mutations, which occur within elements of the repeat. These repeats are of interest as they may encode promoter regions or protein domains. **Ian Korf** and **Michael Brent** (Washington University) jointly presented "Twinscan" which attempts to extend the gene prediction program "Genscan", by extending the model to include homology, with different conservation models for exons, introns UTRs etc. This appears to significantly increase the specificity of "Genscan". They are currently working with EnsEMBL on the mouse genome (**http://genes.cs.wustl.edu**).

Wednesday morning's keynote in the **Methods** section was by **Gunnar von Heijne** (Stockholm University), who gave a very clear talk on *Membrane Proteins: From the Computer to the Bench and Back*. When trying to predict transmembrane

Copyright © 2001 John Wiley & Sons, Ltd.

proteins, topology methods such as "Transmembrane HMM" (TMHMM) are better than hydrophobicity methods. TMHMM has a sensitivity of over 98% when detecting transmembrane proteins, achieving the correct topology 77% of the time. However, if a number of prediction methods are combined then the transmembrane proteins can be predicted with 100% accuracy. These theoretical predictions must be validated by wet lab work, which in turn can help tune the predictive programs. Although, membrane predictions are now reasonably accurate, much information on the helical turning and packing of the amino acids is still missing.

**Daniel Huson** (Celera Genomics) described a Compartmentalized Shotgun Assembler. This involves combining the publicly funded human "clone by clone" BAC" sequence data, with Celera's whole genome shotgun assembly data, to produce a more complete coverage of the genome than either method used separately could produce. **David States** (Washington University) presented a method, at least as accurate as a human expert, to construct physical genomic maps. It can even cope with variable clone length and markers of almost identical molecular weight. **Uri Keich** presented a very interesting and clear talk on behalf of **Pavel Pevzner** (UC San Diego), on assembling "Double-barrelled" data. This assembly method does not use the normal "overlap-layout-consensus" methods utilised by "Phrap", instead the reads are cut into shorter overlapping pieces, which are assembled using a Eulerian Superpath approach, to assign reads to contigs. Repeats are not masked, but rather used to help order contigs, even when there are a number of identical repeats. This method appears to perform very well, giving almost perfect results with all bacterial genomes they looked at.

The only talk on mass spectrometry, was given by **Vineet Bafna** (Celera Genomics) on "SCOPE". "SCOPE" is based on sequence alignment theory to match peptide fragments to peaks produced by Applied Biosystems' new tandem mass spectrometers. They are presently using "SCOPE" to identify 10,000 proteins a day, to an accuracy of 90–99%. **James Borneman** (UC Riverside) introduced an algorithm to choose the minimum probe set to discriminate between populations of microorganisms in the field (even detecting new ones), by DNA fingerprinting of rDNA. **Ziv Bar-Joseph** (MIT) presented an algorithm, which orders the leaves in a tree, or cluster, in a biologically meaningful way. Using yeast cell cycle microarray data they were able to correctly order the genes into cell cycle, and cell cycle group order, which the original clustering method could not do (**http://www.psrg.lcs.mit.edu/clustering/ismb01/optimal.html**). **David Venet** (Université Libre de Bruxelles) gave a talk on a "Direct" method for separating cell types and proportions from heterogeneous samples from colon cancer patients, based on expression profiles, combined with gene annotation.

**Tandy Warnow** (University of Texas, Austin) opened the last session of the conference, which was on **Sequence and Phylogeny**, with a talk on an interesting approach to recreating phylogenies from gene order data rather than gene sequence, although at present the method cannot cope with insertions, deletions and uneven gene content. **Eleazar Eskin** (Columbia University) introduced a method based on mutation matrices, which can be used on any sequence type, to find a probable ancestral sequence (**http://www.cs.columbia.edu/compbio/mca/**). **Luay Nakhleh** (University of Texas, Austin) used a "Dish Covering" algorithm to reconstruct phylogenetic trees. It performed better than Neighbour Joining (NJ) with sequences of over 6000 nucleotides, but NJ performed best with sequences below 4000. **Dirk Husmeier** (Biomathematics and Statistics Scotland) gave the last talk of the conference. He presented an upgrade to "TOPAL", a method for studying recombination. This new measure obtains a tree from a multiple sequence alignment, along which a window is passed and as the window moves new trees are calculated. A difference in the trees indicates differences in the evolution of the sequence on either side of a recombination event (**http://www.bioss.ac.uk/~dirk**).

The conference was brought to a close with a thank you to everyone involved in the organisation of the conference (to which I add my own thanks) and by the presentation of the awards for best poster and talk. **Dana Pe'er** won the best talk, while Boris Lenhard and **Wyeth Wasserman** (Karolinska Institute, Stockholm) won the best poster with *GeneLynx: A Comprehensive and Extensible Portal to the Human Genome*. "GeneLynx" is based on the idea that there should be a central web resource for all human genes, with each gene having a page with links to all the information available on that gene. More details are available from **http://www.genelynx.org**.

ISMB2001 was an excellent conference, with a

Copyright © 2001 John Wiley & Sons, Ltd.

very high standard of both talks and posters, covering a wide range of areas, with a particularly strong representation from the field of microarrays, today's 'hot topic'. So what will next year's hot topic be? Still microarrays, or perhaps mass spectrometry and proteomics?

### References

Goffeau A, *et al.* 1996. Life with 6000 genes. *Science* **274**: 546–547.

Tycowski KT, Shu MD, Steitz JA. 1996. A mammalian gene with introns instead of exons generating stable RNA products. *Nature* **379**(6564): 464–466.

---

The Meeting Reviews of *Comparative and Functional Genomics* aim to present a commentary on the topical issues in genomics studies presented at a conference. The Meeting Reviews are invited; they represent personal critical analyses of the current reports and aim at providing implications for future genomics studies.

*The* **Genomics** *Website at Wiley*

**www.wiley.co.uk/genomics**

The **Genomics** website at Wiley is a **DYNAMIC** resource for the genomics community, offering **FREE** special feature articles and new information **EACH MONTH**.

Find out more about our new journals *Comparative and Functional Genomics,* and *Proteomics.*

Visit the **Library** for hot books in Genomics, Bioinformatics, Molecular Genetics and more.

Click on **Journals** for information on all our up-to-the minute journals, including: *Genesis, Bioessays, Journal of Mass Spectrometry, Gene Function and Disease, Human Mutation, Genes, Chromosomes and Cancer* and the *Journal of Gene Medicine.*

Let the **Genomics** website at Wiley be your **guide** to genomics-related web sites, manufacturers and suppliers, and a calendar of conferences.

WILEY

2380

Copyright © 2001 John Wiley & Sons, Ltd.
